# Children's Departments

**Published:** 1918-10-12

**Authors:** 


### THE WOMAN WORKER'S WORLD.

### Children's Departments.

THE Clerk to the Birmingham Board of Guardians has prepared a most interesting memorandum on that body's six years' work for women and children. Of the latter a little army of 5,439 were committed to the Board's care when three Unions amalgamated in 1912. Between our day and that of Mr. Bumble there is, happily, a great gulf fixed. "A staff of sympathetic women visitors" now exists, and we learn that among those visited are 1,188 children registered under the Act, a rise, indeed, from 488 in 1913 and a fact which in itself shows that care for the individual child is realised as a need, though it is still a future aim that after-care should be bestowed upon *all* children that come through the Guardians' hands. For instance, some forty to fifty children have been adopted under the Acts of 1889 and 1899 and placed out with relations, but systematic visiting of these is not the invariable rule that it should be. Children of families in receipt of outdoor relief are happily much fewer, 1,215 as compared with 3,465 in 1912, and such homes need constant watching to secure an upward in place of a downward trend of character. The truth is that everywhere more voluntary and yet responsible workers are needed for such tasks.

With regard to widows with dependent children, some very good suggestions are made, such as that the disparity between the allowance given for a foster-child and for the mother's own child should be lessened or removed, that effort be made to attach the widow to some helpful social organisation (such encouragement and guidance are often greatly needed by a woman "down on her luck"), and the advice of some officer of the Board in case of an unruly or wayward son. Advance in utilising the services of a woman doctor for pre-natal and post-natal work is desired, as well as in boarding-out and institution provision for delicate children and for crippled ones. For all these purposes, and that of records, which seem curiously deficient, the establishment of a children's department is advised. The point is extremely interesting, raising as it does the whole question of the present sharp severance of the child coming under the Poor Law from others, while at the same time municipalities need a children's department of their own, and the same medical and educational treatment is required by all children.

At the same moment the Board of Education publishes the regulations under which it will give grants to Schools for Mothers, and includes expenditure incurred subsequent to April 1, 1917, for provision of food to expectant and nursing mothers and to underfives who are necessitous, when need is certified by the medical officer. If the school shows itself to be an educational work—that is, one established to train mothers to manage infants and little children—it may carry out this purpose by systematic classes, home visiting, and infant consultations, but the holding of systematic classes is a *sine quâ non*; infant consultations for women who do not attempt them will not be assisted. And grants will be estimated by results, among which the keeping of records is expressly noted, as well as co-ordination with other institutions and authorities. Such grants may amount to one-half of the approved expenditure. It is almost unnecessary to add that such schools must not be run for private profit, but must have a body of managers and an official correspondent.

One of the latest discoveries made in course of finding life-destroying products which incidentally produce a new food is the value of the horse-chestnut. When *acetone* has been extracted a meal remains, which is nourishing for chickens.

